# BPR1J-097, a novel FLT3 kinase inhibitor, exerts potent inhibitory activity against AML

**DOI:** 10.1038/bjc.2011.564

**Published:** 2011-12-20

**Authors:** W-H Lin, W-T Jiaang, C-W Chen, K-J Yen, S-Y Hsieh, S-C Yen, C-P Chen, K-Y Chang, C-Y Chang, T-Y Chang, Y-L Huang, T-K Yeh, Y-S Chao, C-T Chen, J T-A Hsu

**Affiliations:** 1 Institute of Biotechnology and Pharmaceutical Research, National Health Research Institutes, No. 35, Keyan Road, Zhunan, Miaoli County 350, Taiwan; 2 Department of Biological Science and Technology, National Chiao Tung University, Hsinchu, Taiwan

**Keywords:** acute myeloid leukaemia, FLT3, FLT3-ITD, MOLM-13, MV4-11, kinase inhibitor

## Abstract

**Background:**

Activating mutations of Fms-like tyrosine kinase 3 (FLT3) constitute a major driver in the pathogenesis of acute myeloid leukaemia (AML). Hence, pharmacological inhibitors of FLT3 are of therapeutic interest for AML.

**Methods:**

The effects of inhibition of FLT3 activity by a novel potent FLT3 inhibitor, BPR1J-097, were investigated using *in vitro* and *in vivo* assays.

**Results:**

The 50% inhibitory concentration (IC_50_) of BPR1J-097 required to inhibit FLT3 kinase activity ranged from 1 to 10 nM, and the 50% growth inhibition concentrations (GC_50_s) were 21±7 and 46±14 nM for MOLM-13 and MV4-11 cells, respectively. BPR1J-097 inhibited FLT3/signal transducer and activator of transcription 5 phosphorylation and triggered apoptosis in FLT3-driven AML cells. BPR1J-097 also showed favourable pharmacokinetic property and pronounced dose-dependent tumour growth inhibition and regression in FLT3-driven AML murine xenograft models.

**Conclusion:**

These results indicate that BPR1J-097 is a novel small molecule FLT-3 inhibitor with promising *in vivo* anti-tumour activities and suggest that BPR1J-097 may be further developed in preclinical and clinical studies as therapeutics in AML treatments.

Acute myeloid leukaemia (AML) is the most common type of adult leukaemia. It is an aggressive disease that involves rapid growth of abnormal leukaemic cells in the bone marrow, resulting in failure of production of normal blood cells (Dormer *et al*, 1980). Fms-like tyrosine kinase 3 (FLT3), a cell surface receptor belonging to the class III receptor tyrosine kinase family, has a pivotal role in the differentiation and survival of haematopoietic stem cells in the bone marrow (Scheijen and Griffin, 2002; Markovic *et al*, 2005). Fms-like tyrosine kinase 3 is frequently overexpressed and mutated in AML patients and is a major driver in the pathogenesis of AML cancer cells (Stirewalt and Radich, 2003; Kiyoi *et al*, 2005). Activating mutations of FLT3 that result in constitutive FLT3 tyrosine kinase activity lead to activation of downstream signal molecules, including signal transducer and activator of transcription 5 (STAT5), Ras, MAPK, PI3K, and Akt, to subsequently stimulate the survival and proliferation of leukaemic cells (Zhang and Broxmeyer, 1999; Hayakawa *et al*, 2000; Mizuki *et al*, 2000; Brandts *et al*, 2005; Sallmyr *et al*, 2008). Two types of activating FLT3 mutations, namely FLT3-ITD (an internal tandem duplication mutation in the juxtamembrane domain) and FLT3-KDM (a missense mutation at the Asp835 residue within the kinase domain), account for ∼30% of AML patients (Nakao *et al*, 1996; Kiyoi *et al*, 1998; Yamamoto *et al*, 2001; Gilliland and Griffin, 2002a, 2002b). Clinically, these mutations are associated with poor prognosis for AML patients receiving traditional chemotherapy (Thiede *et al*, 2002; Yanada *et al*, 2005). Therefore, activated FLT3 (due to FLT3-ITD and/or FLT3-KDM) is a promising molecular target for AML therapies.

A number of small molecule FLT3 inhibitors have entered clinical trials. These include sorafenib (BAY 43-9006), sunitinib (SU11248), midostaurin (PKC412), lestaurtinib (CEP-701), tandutinib (MLN518), ABT-869, KW-2449, and AC220 (Wong *et al*, 2009; Kindler *et al*, 2010; Wiernik, 2010). However, approval of these agents for FLT3-associated diseases is still challenging, which was suspected to be due to the failure to fully inhibit FLT3 in tumours and undesirable drug properties (Ustun *et al*, 2009). The purpose of this study was to characterise the pharmacological profile of a novel FLT3 kinase inhibitor, BPR1J-097 (*N*1-(3-3-[(phenylsulphonyl)amino]phenyl-1*H*-5-pyrazolyl)-4-(4-methylpiperazino) benzamide) (Figure 1), that was discovered by a rational design strategy. BPR1J-097 with a novel sulphonamide pharmacophore exhibits potent FLT3-inhibitory activity and has potent growth-inhibitory effects on FLT3-ITD leukaemic cells. According to our SAR studies, we found that sulphonamide pharmacophore preferred at meta-position of the phenyl ring, and that replacement the sulphonamide group of BPR1J-097 with the urea group resulted in a significant loss in cellular potency (data not shown). Inhibition of FLT3 resulted in blockage of FLT3 and STAT5 phosphorylation and triggered apoptosis in cancer cells relying on FLT3 signalling for survival. In addition, BPR1J-097 showed favourable pharmacokinetic properties and significant dose-dependent tumour reduction in FLT3-ITD murine xenograft models.

These results demonstrate the potential of BPR1J-097 as a therapeutic candidate for treatment of AML patients. Further preclinical and clinical studies in AML patients are warranted.

## Materials and methods

### Reagents

The FLT3 inhibitors BPR1J-097 and ABT-869 were synthesised by our laboratory. Sorafenib and PKC412 were obtained from Calbiochem (Darmstadt, Germany). The recombinant human FLT3 ligand (T27-P185) was purchased from R&D Systems (Minneapolis, MN, USA). The anti-phospho and total (FLT3, STAT5, and Erk1/2) (Cell Signaling Technology, Beverly, MA, USA), anti-cleaved poly (ADP-ribose) polymerase (Cell Signaling Technology), and anti-caspase 3 (Imgenex, San Diego, CA, USA), anti-*β*-actin (Santa Cruz Biotechnology, Santa Cruz, CA, USA) antibodies were purchased for western blotting analysis. The N-terminal GST-tagged human FLT3 (residues Y567-S993), VEGFR1 (residues R781-I1338), and VEGFR2 (residues V789-V1356), expressed in *Sf9* insect cells, were purified for kinase assay (Lin *et al*, 2008). The preparation of recombinant Aurora A (residues R781-I1338) was described in a previous study (Coumar *et al*, 2008). Aurora B (Upstate, Billerica, MA, USA) was purchased for biochemical kinase assay. Human full-length FLT3 expression clone (pCMV6-Entry-FLT3-WT, RC211459) was purchased from OriGene (Rockville, MD, USA). The pCMV6-Entry-FLT3-ITD plasmid was constructed by introducing the RT–PCR fragment, generated by 5′-TATGCAACAATTGGTGTTTGTCTCC-3′ and 5′-TACGCGTCGAATCTTCGACCTGAGAGCCTGC-3′ primers with MV4-11 cDNA templates, into pCMV6-Entry-FLT3-WT MfeI- and MluI-digested fragments. The pCMV6-Entry-FLT3-D835Y plasmid was constructed by mutagenic primers (5′-TGGATTGGCTCGATATATCATGAGTGA-3′ and 5′-TCACTCATGATATATCGAGCCAATCCA-3′) and pCMV6-Entry-FLT3-WT as template using the QuikChange II Site-Directed Mutagenesis Kit (Agilent Technologies, Santa Clara, CA, USA) according to the manufacturer's recommendations.

### Cell lines

MOLM-13 cells were purchased from the Deutsche Sammlung von Microorganismen und Zellkulturen GmbH (Braunschweig, Germany). RS4;11, MV4-11, U937, and K562 cell lines were obtained from the American Type Culture Collection (ATCC, Manassas, VA, USA). All cell lines were grown in RPMI 1640 (Invitrogen, Carlsbad, CA, USA) with 10% fetal bovine serum (FBS) (Fisher Scientific, Pittsburgh, PA, USA). HEK293T- and FLT3-transfected HEK293T cells were cultured in DMEM (Invitrogen) medium with 10% FBS.

### Biochemical kinase assays

The FLT3 Kinase-Glo kinase assays were carried out in 96-well plates at 30°C for 4 h in a final volume of 50 *μ*l, including 25 mM Tris pH 7.4, 10 mM MgCl_2_, 4 mM MnCl_2_, 1 mM DTT, 0.02% Triton X-100, 0.01% BSA, 1 *μ*M ATP, 20 *μ*M peptide (GGMEDIYFEFMGGKKK), 75 ng recombinant FLT3 proteins, and test compound at the indicated concentration. The VEGFR1 or VEGFR2 kinase assay was carried out in 96-well plates with tested compound in a final volume of 50 *μ*l reaction at 30°C for 2 h with the following components: 25 mM HEPES pH 7.4, 10 mM MgCl_2_, 4 mM MnCl_2_, 0.5 mM Na_3_VO_4_, 2 mM DTT, 0.02% Triton X-100, 0.01% BSA, 1 *μ*M ATP, 2 *μ*M polyGlu4:Tyr peptide, 100 ng recombinant VEGFR1 or VEGFR2 protein. Aurora kinase A and Aurora kinase B assays were performed as reported by us in an earlier study (Coumar *et al*, 2008). After incubation, 50 *μ*l Kinase-Glo Plus Reagent (Promega, Madison, WI, USA) was added and incubated at 25°C for 20 min. A 70 *μ*l aliquot of each reaction mixture was transferred to a black microtiter plate and the luminescence was measured on a Wallac Vector 1420 multilabel counter (Perkin-Elmer, Shelton, CT, USA). Each IC_50_ value was determined by three different experiments. Kinase inhibition profiling and FLT3-D835Y-inhibitory activity were determined by Invitrogen SelectScreen kinase profiling service.

### Cellular proliferation assays

Proliferation assays were performed by seeding 10 000 cells per well in a 96-well culture plate. After 16 h, cells were then treated with vehicle or test compounds at various concentrations of the tested compound in medium for 72 h. Cell viability was quantitated using the MTS method (Promega) according to the manufacturer's recommended protocol. The results were determined by measuring absorbance at 490 nm using a plate reader (Victor2; Perkin-Elmer). The GC_50_ value was defined as the amount of compound that caused 50% reduction in cell viability in comparison with DMSO-treated (vehicle) control and was calculated using Prism version 4 software (GraphPad, San Diego, CA, USA).

### Western blotting

Cells were lysed in lysis buffer (50 mM Tris, pH 8.0, 150 mM NaCl, 1% Triton X-100, 0.5% sodium deoxycholate, 0.1% SDS, 1 mM sodium orthovanadate, 1 mM PMSF, and 1 mM DTT). Protein lysates were resolved in SDS–PAGE and then transferred onto a polyvinylidene difluoride membrane (Millipore, Bedford, MA, USA). Membranes were immunoblotted with appropriate antibodies and reacted with the SuperSignal reagent (Pierce, Rockford, IL, USA), followed by exposure to X-ray film.

### Pharmacokinetic studies

Male Sprague–Dawley rats weighing 300–400 g each (8–12 weeks old) were obtained from BioLASCO (Taiwan Co., Ltd, Ilan, Taiwan). Animals were surgically prepared with a jugular-vein cannula one day before dosing and fasted overnight (for approximately 18–20 h) before dosing. Water was available *ad libitum* throughout the experiment. Food was provided at 4 h after dosing. Single 3.4 mg kg^−1^ dose of BPR1J-097, as a PEG400/water (80/20, v/v) solution, was separately administered to groups of 3 rats each intravenously (i.v.) by a bolus injection through the jugular-vein cannula. Each animal received 1 ml of the dosing solution per kg of body weight i.v. At 0 (before dosing), 2, 5, 15, and 30 min and at 1, 2, 4, 6, 8, and 24 h after dosing, a blood sample (0.15 ml) was collected from each animal through the jugular-vein cannula and stored in ice (0–4°C). Immediately after collecting the blood sample, 150 *μ*l of physiological saline (containing 30 Units of heparin per ml) was injected into the rat through the jugular-vein cannula. Plasma was separated from the blood by centrifugation (14 000 **g** for 15 min at 4°C in a Beckman Model AllegraTM 6R centrifuge) and stored in a freezer (−20°C). All samples were analysed for the parent drug by LC-MS/MS. Data were acquired through selected reaction ion monitoring. Plasma concentration data were analysed with non-compartmental method.

### Subcutaneously xenograft tumour models

Male nude mice (Nu-Fox1nu) of 8 weeks of age were purchased from BioLASCO (Taipei, Taiwan, R.O.C.). Nude mice (*n*=5–7 per group) were inoculated subcutaneously with MOLM-13 (1 × 10^6^ per flank) or MV4-11 cells (5 × 10^6^ per flank). All human cancer cells were detected as free of Mycoplasma spp before they were injected into animals. When the tumour size reached 100–200 mm^3^, animals were grouped and treated with BPR1J-97 at various doses in a 2-week treatment period as indicated. Animals were treated with BPR1J-097 (10 and 25 mg kg^−1^, i.v.) or vehicle as control at once daily for 5 days per week for 2 weeks. Tumour volumes were measured and calculated with the formula length × width^2^/2 after initiation of treatments. Tumour size and animal body weight were measured twice a week after tumour cell inoculation. At the end of the study, animals were killed by carbon dioxide inhalation followed by cervical dislocation. The significant difference between drug treatment and vehicle control were analysed using one-way *ANOVA* and Student–Newman–Keuls test. The level of a statistical significance was set at *P*<0.05. The uses and experimental procedures in animals were approved by the IACUC (Institutional Animal Care and Use Committee) of the National Health Research Institutes and met the standards required by the UKCCCR (United Kingdom Coordinating Committee on Cancer Research) guidelines (Workman *et al*, 2010).

## Results

### BPR1J-097 is a potent inhibitor of FLT3 activity

**Figure 1:**
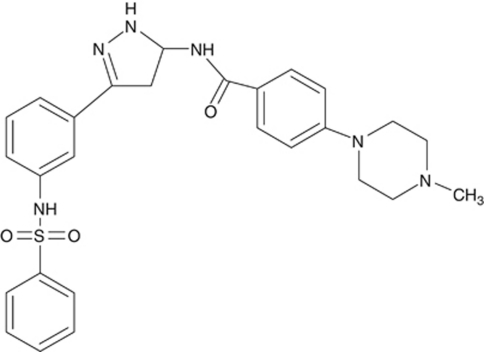
Chemical structure of BPR1J-097 (*N*1-(3-3-[(phenylsulphonyl)amino]phenyl-1*H*-5-pyrazolyl)-4-(4-methylpiperazino) benzamide).

BPR1J-097 is a novel compound with a novel sulphonamide pharmacophore (Figure 1). BPR1J-097 exhibits potent FLT3-inhibitory activity and has potent growth-inhibitory effects on FLT3-ITD leukaemic cells. As shown in Table 1, BPR1J-097 potently inhibited wild-type FLT3 (FLT3-WT) activity with an IC_50_ of 11±7 nM. In comparison, the IC_50_ of ABT-869 was 17±7 nM as measured in this study. The IC_50_ value of ABT-869 for inhibition of FLT3-WT was 4 nM as previously reported by Shankar *et al* (2007). BPR1J-097 specifically targets FLT3 kinase with weaker inhibitory activity towards related kinases such as FLT1 (VEGFR1) and KDR (VEGFR2) (Table 2). In a screening assay for kinase inhibition specificity, 59%, and 91% of FLT1 and KDR activities, respectively, were inhibited by BPR1J-097 at 1 *μ*M. Subsequently, the IC_50_s of BPR1J-097 were determined to be 211 and 129 nM for FLT1 and KDR, respectively. As some FLT3 inhibitors were also found to be potent inhibitors for Aurora kinases (Harrington *et al*, 2004; McLaughlin *et al*, 2010), due to the high degree of similarity in their ATP-binding pockets (Pollard and Mortimore, 2009), the IC_50_s of BPR1J-097 were also determined for Aurora A and B kinases. It is interesting to note that the IC_50_s of BPR1J-097 were 340 and 876 nM for Aurora A kinase and Aurora B kinase, respectively. Overall, BPR1J-097 is a quite specific FLT3 inhibitor.

### BPR1J-097 inhibits the phosphorylation of FLT3 and STAT5 in cells

**Figure 2:**
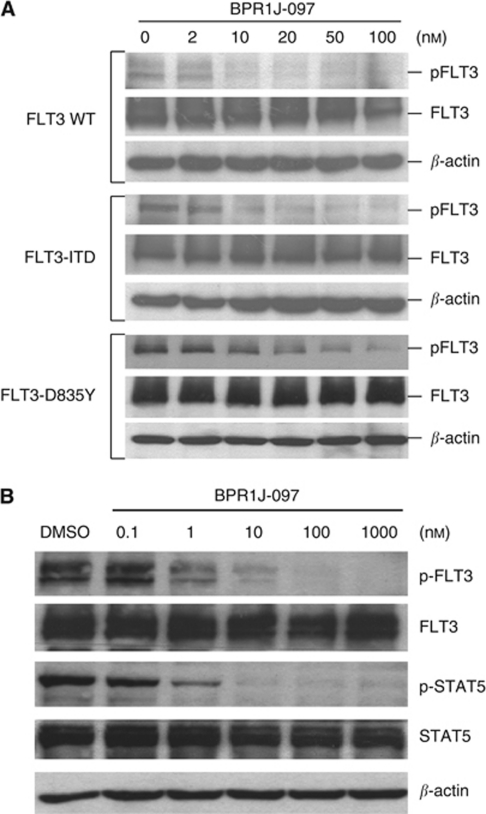
BPR1J-097 inhibits FLT3-dependent signalling. (**A**) HEK 293T cells were transfected with FLT-WT-, FLT3-ITD-, or FLT3-D835Y-expressing plasmids for 24 h and then incubated with various concentrations of BPR1J-097 for 2 h. (**B**) MV4-11 cells, which are homozygous for FLT-ITD, were treated with BPR1J-097 at the indicated concentrations for 2 h. The phosphorylation states of FLT3 and STAT5 were evaluated by western blot.

To examine whether BPR1J-097 was able to inhibit the phosphorylation of FLT3, HEK293T cells were transfected with plasmids encoding the FLT3-WT or mutants FLT3 (FLT3-ITD, FLT3-D835Y). 293T cells were treated with BPR1J-097 at various concentrations for 2 h and then the FLT3 ligand (50 ng ml^−1^) was added for 5 min to prepare cell lysates for western analysis. As shown in Figure 2A, phosphorylation of all FLT3-WT, FLT3-IDT, and FLT3-D835Y were inhibited by BPR1J-097 at a concentration as low as 10 nM. The constitutive activation of STAT5 has a pivotal role in FLT3-ITD leukaemia cell expansion and survival (Hayakawa *et al*, 2000). To characterise the effects of BPR1J-097 on the FLT3 signalling pathway in FLT3-driven cells, MOLM-13 and MV4-11 cells were cultured in the presence of different concentrations of BPR1J-097 for 2 h, and inhibition of the phosphorylation of FLT3 and STAT5 was examined by western blot analysis. As shown in Figure 2B, BPR1J-097 suppressed the phosphorylation of FLT3 and STAT5 in a dose-dependent manner. The observed IC_50_ was ∼10 nM for FLT3-ITD activity and 1 nM for STAT5 phosphorylation.

### BPR1J-097 inhibits proliferation of FLT3-dependent cells

Potent anti-proliferative activity of BPR1J-097 was observed in FLT3-driven MOLM-13 and MV4-11 AML cell lines containing the FLT-ITD-activating mutation. The IC_50_ values of BPR1J-097 on MOLM-13 and MV4-11 cells were 21±7 and 46±14, respectively. In contrast, RSV4;11, U937, and K562 cells the growth of which is independent of FLT3 signalling (Yee *et al*, 2002; Pallis *et al*, 2003; Odgerel *et al*, 2008), were weakly inhibited by BPR1J-097 (Table 3). Thus, BPR1J-097 is a potent inhibitor for the proliferation for FLT3-driven cells and BPR1J-097 is not generally cytotoxic.

### BPR1J-097 induces apoptosis in FLT3-ITD leukaemic cells

**Figure 3:**
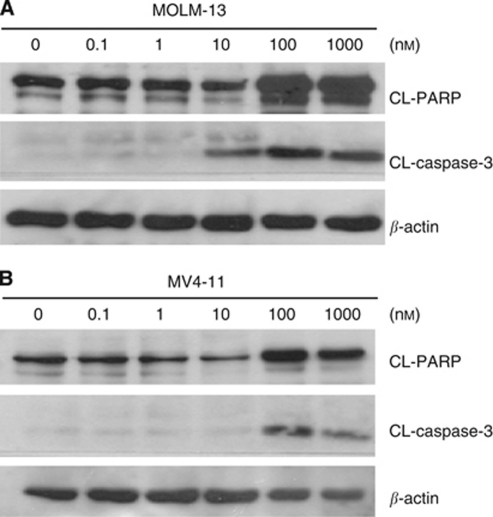
BPR1J-097 induces apoptosis in MOLM-13 and MV4-11 cells. Western blotting showed that BPR1J-097 was able to induce apoptosis in FLT3-driven AML cells. MOLM-13 (**A**) and MV4-11 (**B**) cells were treated with BPR1J-097 at the indicated concentrations for 48 h, and then cell lysates were subjected to western blot analysis with cleavage of poly(ADP-ribose) polymerase (PARP) and caspase 3.

Fms-like tyrosine kinase 3 inhibition would trigger apoptosis in cells dependent on FLT3 for cell growth and survival. We then evaluated whether BPR1J-097 induced cell death in relevant cells. Both MOLM-13 and MV4-11 cells were treated with BPR1J-097 at different concentrations for 48 h before western blot analysis. The emergence of active caspase-3 (CL-caspase-3) was observed in MOLM-13 cells treated with BPR1J-097 at 10 nM (Figure 3A). However, the effect of BPR1J-097 seems to be weaker in MV4-11 cells as CL-caspase-3 was not evident until 100 nM of BPR1J-097 was applied to treat cells (Figure 3B). It seemed that the anti-proliferation activity of BPR1J-097 is more pronounced in MOLM-13 than in MV4-11 cells.

### Pharmacokinetic parameters of BPR1J-097

**Figure 4:**
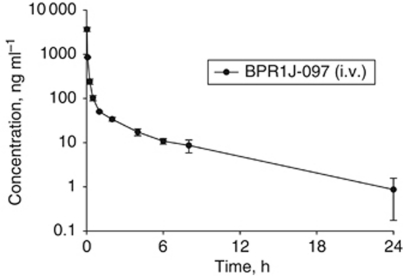
Pharmacokinetic profile of BPR1J-097 in rats. A single intravenous bolus dose (3.4 mg kg^−1^) of BPR1J-097 was administered to the adult male Sprague–Dawley rats (*n*=3). Data illustrate mean values (*n*=3)±s.d. for each time of plasma concentrations of BPR1J-097.

To assess the pharmacokinetic properties of BPR1J-097, the plasma concentration of BPR1J-097 over a 24-h period was measured after a single i.v. administration (Figure 4 and Table 4). BPR1J-097, after a 3.4 mg kg^−1^ i.v. dose administered to rats, achieved a maximum plasma level maximum concentration of 7.1 *μ*M (3670 ng ml^−1^) at 2 min of dosing and, at 24 h after dosing, the estimated BPR1J-097 plasma concentration remained at a concentration of 1 ng ml^−1^ (1.9 nM). The total body clearance was 102.4±9.8 ml min^−1^ per kg and the volume of distribution at the steady state (*V*_ss_) was 15.5±4.8 l kg^−1^ for BPR1J-097 in rats. The apparent plasma half-life was ∼4.5 h.

### *In vivo* tumour growth-suppressing activities of BPR1J-097

**Figure 5:**
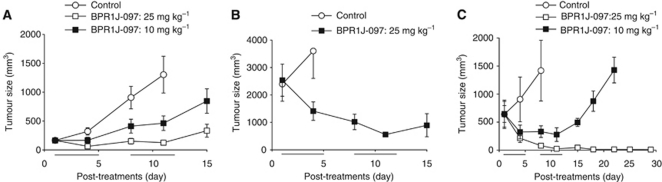
Anti-tumour activity of BPR1J-097 against FLT3-driven leukaemia tumour growth in nude mice. (**A**) *In vivo* anti-tumour effect of BPR1J-097 in the MOLM-13 xenograft nude mice model. Growth of the tumour xenograft was inhibited by BPR1J-097 (10 or 25 mg kg^−1^, i.v.); *P*<0.05 compared with vehicle treatment. (**B**) The subcutaneously growing MOLM-13 tumour of large size (>2000 mm^3^) in nude mice could be significantly shrunk by BPR1J-097 (25 mg kg^−1^, i.v.). (**C**) BPR1J-097 at 10 and 25 mg kg^−1^ (i.v.) showed significant suppression of MV4-11 tumour growth; *P*<0.05, *n*=5–6 per group. Mean tumour volumes±s.e.m. are shown. Vehicle (○), 10 mg kg^−1^ (▪), 25 mg kg^−1^ (□).

To examine whether BPR1J-097 exhibited anti-tumour activity *in vivo*, MOLM-13 or MV4-11 cells were subcutaneously implanted into nude mice. In the MOLM-13 model, tumours were allowed to grow to a size of approximately 100–200 mm^3^. After i.v. administration of mice with BPR1J-097 at two cycles of 10 or 25 mg kg^−1^, a clear dose-dependent anti-tumour effect was observed (Figure 5A). Tumours in mice treated with 25 mg kg^−1^ per day stopped growing. After the treatment was terminated, tumours continued to grow. ABT-869 was shown to be unable to shrink the MOLM-13 xenograft tumours (Shankar *et al*, 2007). To examine whether BPR1J-097 can shrink established tumour mass, the MOLM-13 xenograft was allowed to grow to a size over 2000 mm^3^. In contrast to ABT-869, BPR1J-097 (25 mg kg^−1^) showed a significant tumour shrinkage effect on the subcutaneously growing MOLM-13 tumours in a size of >2000 mm^3^ (Figure 5B). Tumours started to grow after the termination of BPR1J-097 treatment. Compared with ABT-869, BPR1J-097 seemed to be more efficacious in the MOLM-13 xenograft model. Furthermore, BPR1J-097 (10 and 25 mg kg^−1^) also produced a dose-dependent growth reduction and shrinkage of another model using MV4-11 cells. It is noted that a prolonged disappearance of MV4-11 tumours was observed in mice treated with BPR1J-097 at 25 mg kg^−1^ (Figure 5C). There was little (3%) or no body weight loss of BPR1J-097-treated nude mice during the observation periods in these *in vivo* studies. It is interesting to note that although BPR1J-097 was able to trigger more apoptosis in MOLM-13 cells than in MV4-11 cells (Figure 3A), BPR1J-097 seemed more effective for MV4-11 than for MOLM-13 xenograft tumours (Figure 5C). Further studies are required to elucidate the underlying mechanism.

## Discussion

Aberrant regulation of tyrosine kinase has been implicated as a causal factor in tumourigenesis, cancer progression, and drug resistance in many tumours. Pharmacological inhibition of dysregulated kinases is an attractive therapeutic approach for treatment of cancers. Imatinib, a Bcr-Abl kinase inhibitor, and gefitinib, an EGFR tyrosine kinase inhibitor, showed remarkable efficacy in chronic myelogenous leukaemia and in a subset of non-small cell lung cancer, respectively (Cohen *et al*, 2003; Schiffer, 2007). Fms-like tyrosine kinase 3 is the tyrosine kinase that is commonly found in an aberrant form, including overexpression and activating mutant forms, to contribute to AML oncogenesis. Two principal types of aberrant FLT3-activating mutations, namely FLT3-ITD and FLT3-KDM, were shown to account for almost 30% of AML patients, and these mutations are associated with poor prognosis in patients receiving chemotherapy. Therefore, FLT3 is an attractive therapeutic target for discovery of novel therapeutics for AML treatment.

Several existing drugs or experimental compounds with inhibitory activity against FLT3 kinase are being evaluated for treatment of AML patients (Weisberg *et al*, 2002; O’Farrell *et al*, 2003; Griswold *et al*, 2004; Smith *et al*, 2004; Stone *et al*, 2005; Auclair *et al*, 2007; Shankar *et al*, 2007; Shiotsu *et al*, 2009; Zarrinkar *et al*, 2009). Clinical studies have shown that treatment with a single FLT3 inhibitor does not yield therapeutic benefits in AML patients, suggesting that they may not be optimal to fully inhibit FLT3 in tumours. Alternatively, current FLT3 inhibitors may not be active enough to inhibit pre-existing drug-resistant FLT3 or new drug-resistant FLT3 mutants, which can be readily acquired after initiation of treatment (Breitenbuecher *et al*, 2009a, 2009b). Thus, continuous efforts are warranted to design and discover novel and more effective inhibitors of FLT3.

Through rational design, we discovered a potent FLT3 inhibitor, BPR1J-097, which effectively inhibits FLT3 activity *in vitro* and *in vivo.* The inhibitory activity of BPR1J-097 was characterised using various assays including *in vitro* kinase activity, cell-based phosphorylation of FLT3 and a major downstream signalling modulator, STAT5, and proliferation of FLT3-driven leukaemic cells under *in vitro* and *in vivo* conditions. We found that BPR1J-097 potently inhibits FLT3 activity in the *in vitro* kinase assay compared with other FLT3 inhibitors such as ABT-869, sorafenib, and PKC412 (Table 1). In addition, BPR1J-097 inhibited proliferation of FLT3-driven cells (MOLM-13 and MV4-11), but not FLT3-independent cells (U937, RSV;11, and K562), with equal or better potency and selectivity than other FLT3 inhibitors (Table 3). To assess the FLT3-ITD-inhibitory activity of BPR1J-097, we measured FLT3 phosphorylation in transfected 293T-FLT3-ITD and FLT3-ITD-homozygous MV4-11 cells. Results showed that BPR1J-097 decreased FLT3-ITD phosphorylation levels with an observed IC_50_ of approximately 1–10 nM. Transfected cells with the FLT3-D835Y mutant was also inhibited by BPR1J-097 with similar IC_50_ values in 293T-FLT3-ITD cells. Proliferation and aberrant FLT3 signalling were both inhibited by BPR1J-097, with an IC_50_ of 10 nM. Treatment of FLT3-driven cell lines with BPR1J-097 led to induction of apoptosis.

The maximum achievable plasma concentration of BPR1J-097 after a single dose of 3.4 mg kg^−1^ administration to rats is >645-fold above the IC_50_ for FLT3-ITD inhibition in the biochemical and cellular assays. Even at 24 h after single dosing, plasma levels of BPR1J-097 were high enough for complete inhibition of FLT3-ITD. In addition, the high *V*_ss_ indicated that the distribution of BPR1J-097 into a deep, including tumour, tissue compartment is expected. These good pharmacokinetic properties indicated that BPR1J-097 dosing once a day is sufficient for continuous inhibition of FLT3 activity in rats or mice.

To evaluate the anti-tumour efficacy of BPR1J-097 *in vivo*, we used MOLM-13 and MV4-11 murine xenograft models. Results showed that BPR1J-097 administration resulted in significant tumour regression in those two models. In a previous report, ABT-869 was not able to produce regressions of tumours in the MOLM-13 model (Shankar *et al*, 2007). In contrast, in our study, BPR1J-097 reduced the size of tumours even when they were allowed to grow to a size of >2000 mm^3^ in the MOLM-13 xenograft tumour model (Figure 5B). In the MV4-11 model, BPR1J-097 completely eliminated tumours after two cycles of treatment (Figure 5C). The precise mechanism of its strong anti-tumour efficacy in animals will require further investigation.

In conclusion, these data demonstrate that BPR1J-097 exhibits potent FLT3-inhibitory activity in both *in vitro* and *in vivo* assays, excellent selectivity among the kinases examined, and favourable pharmacokinetic properties. Further studies of the clinical features of BPR1J-097 will be required to evaluate whether BPR1J-097 may have therapeutic benefit for AML patients.

## Figures and Tables

**Table 1:** FLT3 kinase-inhibitory activity of BPR1J-097

***In vitro* kinase inhibition IC_50_, nM**	
BPR1J-097	11±7
ABT-869	17±7
Sorafenib	44±9
PKC412	37±5

Abbreviation: FLT3=Fms-like tyrosine kinase 3.

Each IC_50_ determination was performed with eight concentrations by kinase-Glo assay. Data represent mean±s.d. from three different experiments.

**Table 2:** Specificity of kinase inhibition of BPR1J-097

**Kinase**	**Percentage of inhibition**	**IC_50_^b^, nM**
FLT3	96	11
FLT3-D835Y	99	3^c^
FLT1 (VEGFR1)	59	211
KDR (VEGFR2)	91	129
PDGFRA (PDGFR-*α*)	80	ND
TEK (Tie2)	61	ND
KIT	49	ND
AURKA (Aurora A)	80	340
AURKB (Aurora B)	79	876
AURKC (Aurora C)	26	ND
AMPK A1/B1/G1	82	ND
SRC	81	ND

Abbreviations: FLT3=Fms-like tyrosine kinase 3; ND=not determined; PDGFR=platelet-derived growth factor receptor; VEGFR=vascular endothelial growth factor receptor.

a Inhibition of kinase at 1 *μ*M, carried out by Invitrogen SelectScreen kinase profiling service.

b IC_50_ determination was performed by kinase-Glo assay.

c IC_50_ determination was performed by Invitrogen SelectScreen kinase assay.

**Table 3:** Growth-inhibitory activities of BPR1J-097 on various leukaemia cell lines

		**Proliferation GI_50_, nM**			
**Cell lines**	**Characterisation**	**BPR1J-097**	**ABT-869**	**Sorafenib**	**PKC412**
MOLM-13	AML-FLT3-ITD (heterozygous)	21±7	38±14	82±37	55±18
MV4-11	AML-FLT3-ITD (homozygous)	46±14	82±17	43±10	38±12
U937	AML-FLT3-negative	>20 000	>18 000	3350±1200	1400±900
RSV;11	ALL-WT-FLT3 (homozygous)	9400±700	9200±2700	9300±1200	400±100
K562	CML-Bcr-Abl FLT3 –negative	>20 000	>20 000	7300±2700	>20 000

Abbreviations: ALL=acute lymphoblastic leukaemia; AML=acute myelocytic leukaemia; CML=chronic myelogenous leukaemia; WT=wild-type.

Data represent means±s.d. from three different experiments.

**Table 4:** Pharmacokinetic parameters of BPR1J-097 in rats

	**Rat IV (dose: 3.4 mg kg^−1^)**			
**Compound**	**T_1/2_ (h)**	**CL (ml min^−1^ per kg)**	***V*_ss_ (l/kg)**	**AUC_(0–24)_ (ng ml^−1^ × h)**
BRP1J-097	4.5±1.5	102.4±9.8	15.5±4.8	551±51

Abbreviations: AUC=area under the curve; CL=Clearance; *V*_SS_=volume of distribution at the steady state.
